# Understanding factors affecting engagement with food-related services in areas of deprivation: A mixed-methods approach

**DOI:** 10.1177/13591053251404162

**Published:** 2026-01-08

**Authors:** Abigail Stephen, Oana Petre, Janet Kyle, Frank Thies, Julia L. Allan

**Affiliations:** 1 University of Aberdeen, UK; 2 University of Stirling, UK

**Keywords:** service engagement, COM-B, stigma, thematic analysis, mixed methods

## Abstract

Poor dietary health is disproportionately higher in low-income adults, who often face multiple barriers to accessing affordable, nutritious food. Food-related services aim to address these challenges, but engagement remains inconsistent. Understanding the drivers of (non)engagement is critical to improving service design and delivery. This mixed-methods study explored factors influencing engagement with food-related services among low-income adults living in deprived areas. A cross-sectional survey (*n* = 77) measured capability, opportunity, and motivation to engage with services using the COM-B (capability, opportunity, motivation – behaviour) model. Semi-structured interviews (*n* = 20) were analysed thematically to explore service users’ lived experiences. Survey findings indicated that perceived capability and opportunity, but not motivation, were positively associated with service engagement. In contrast, qualitative data revealed that motivation was often present but disrupted by external barriers, such as stigma and unmet needs. Future interventions should prioritise equitable access, dignity-preserving practices, and responsiveness to user needs to reduce barriers and enhance engagement.

## Introduction

Dietary health behaviours are influenced by a range of structural and individual factors, such as food access, cost, and availability (Williamson et al., 2017). Although dietary behaviours are modifiable, adopting a healthy diet is particularly challenging for individuals living in deprived areas (Scott et al., 2018). As the cost of ‘healthy’ food items continues to outpace the cost of ‘unhealthy’ foods in high-income countries, a nutritious diet is becoming increasingly challenging for people in deprived areas to attain (Hoenink et al., 2022; Jones et al., 2014; Scott et al., 2018). The ongoing cost-of-living crisis continues to exacerbate this issue (Hoenink et al., 2024), leading to a high prevalence of food insecurity in deprived communities (Dowler and O’Connor, 2012).

In response, a range of food-related services and community-based interventions have been developed to support individuals in low-income and deprived areas. Typically situated within local charities, community organisations, or national networks, these services are well established in deprived communities (Sosenko et al., 2013; Williams and May, 2022) and support people to access food despite financial constraints (Caplan, 2016; Everson-Hock et al., 2013); services include food banks, community cafés, and cooking or nutrition programmes (Caplan, 2016; Everson-Hock et al., 2013). These interventions often offer a combination of direct food provision, sharing of health information, and teaching of skills like cooking or budgeting (Bush et al., 2014; Dombrowski et al., 2020; Garcia et al., 2020; Loopstra and Tarasuk, 2013).

Despite their availability, engagement with food-related services is inconsistent and often suboptimal with some evidence suggesting that as few as 20% of those in need of food banks will access them (Power et al., 2023). Attrition rates in diet and health related interventions are typically higher for participants from deprived areas (Birch et al., 2022). Suboptimal access and engagement with support services may have widespread negative impacts on wellbeing and dietary health, and in turn exacerbate health problems and health inequalities (Garthwaite et al., 2015).

It remains unclear why uptake of food support services in the community is low. Understanding engagement with food-related services is complex, as these services vary widely in their focus, from food provision (e.g. food banks) to behaviour change based approaches (e.g. healthy eating classes). Some research suggests it is due to poor service provision (Dowler and O’Connor, 2012; Garthwaite et al., 2015) and other studies have suggested that logistical challenges (e.g. transport, childcare), stigma, and psychological disengagement may affect engagement (Michie et al., 2009; Power et al., 2023). Identifying the factors influencing engagement would help ensure that any novel food-related services can be developed, tailored and delivered appropriately.

To date, relatively few studies have applied behavioural models to understand food service engagement. It may be better understood through theoretical models from health psychology, such as the COM-B (capability, opportunity, motivation – behaviour) model (Michie et al., 2011) which proposes that behaviour is driven by three core components: capability, opportunity, and motivation. Applying this model can help identify key determinants of engagement and inform targeted strategies to improve service uptake. Understanding the drivers of engagement also requires capturing the lived experience of potential participants, to gather valuable insights into factors affecting their need, ability, or willingness to use these services (Neve et al., 2021). Living in deprivation and experiencing food insecurity is a deeply personal lived experience, shaped by structural inequalities, stigma, and individual circumstances (Douglas et al., 2020).

The present study aims to investigate drivers of food service engagement within community settings characterised by high levels of multiple deprivation, as defined by the Scottish Index of Multiple Deprivation (Scottish Government, 2020b).

## Materials and methods

A mixed-methods approach was implemented. First, a cross-sectional quantitative survey based on the COM-B model was developed and administered to the intended beneficiaries of food related services in deprived communities to understand how capability, opportunity and motivation are related to engagement. Then semi-structured qualitative interviews were conducted with a different sample of intended beneficiaries to understand the factors that affect their ability and willingness to engage with food services.

### Setting

This study was conducted in Aberdeen, a city with a diverse socioeconomic profile and experiencing significant levels of multiple deprivations. Participants were recruited via convenience and snowball sampling methods; posters and flyers advertising the study were distributed in different settings located within deprived communities (including charity projects, shops, food banks, social cafes). Interviews and surveys were typically conducted/administered in private rooms within community centres. Areas of deprivation were identified using the Scottish Index of Multiple Deprivation (Scottish Government, 2020b).

#### Phase 1 – COM-B survey

The protocol for this phase of the study was pre-registered on the Open Science Framework (DOI: [10.17605/OSF.IO/2VT84]) and was granted ethical approval by the Rowett Institute’s Ethics Council in June 2024. All participants gave informed consent and received a £10 grocery shop voucher as a gratuity.

Between June and July 2024, participants were invited to complete an anonymous, self-administered survey to assess their self-reported engagement with community food-related interventions and the factors that affect engagement. An a priori power calculation with G*Power, version 3.1.9.7, indicated a minimum sample size of *n* = 60 was required to detect a small-medium effect (α = 0.05, power = 0.80), based on the protocols of previous COM-B studies (Howlett et al., 2021; Huynh et al., 2023). The researchers aimed to recruit an additional 20% (*n* = 13) to compensate for incomplete submissions.

Demographic characteristics (age, gender, ethnicity, education, and household) were measured with a short eight-item questionnaire. Food insecurity level was assessed with a three-item self-reported food insecurity scale, taken from the Scottish Health Survey (Scottish Government, 2020a).

Capability, opportunity, motivation and behaviour were measured via an 11-item questionnaire (Table 1) developed by AS and JA using published descriptions of COM-B constructs (Michie et al., 2011). Responses were scored on a 5-point Likert-scale from 1 (strongly agree) to 5 (strongly disagree), then averaged for each construct and reverse scored for simplicity so a higher score indicated greater capability/opportunity/motivation/behaviour. To maximise accessibility, items were worded using simple/lay language, surveys were provided in both digital and print format, and surveys were conducted within community venues.

**Table 1.** Items from the survey, corresponding to each of the COM-B model components.

Capability	• I know what community food services there are in my area
	• I can access community food services if I need to
	• At times when I have lots going on or am really tired, I am still able to access these services
Opportunity	• It’s easy for me to access these services
	• My friends and family think I should use these services
Motivation	• Other people like me use these services
	• I am confident that I can use these services
	• Using these services is helpful to me
	• I want to use these services
	• I feel bad when I use these services
Behaviour	• I regularly use services in my local area that offer support for healthy eating

Descriptive analyses were used to summarise participants’ characteristics and level of food insecurity. Multiple linear regression was used to predict engagement with community food-related interventions from capability, opportunity, and motivation. Analysis was conducted using SPSS (IBM Corp, 2023)

#### Phase 2 – Semi-structured interviews

This phase of the study was granted ethical approval by the Rowett Institute’s Ethics Council in November 2023. Each participant provided informed consent and were paid £20 as compensation for their time.

Semi-structured qualitative interviews were used to explore service participants’ experiences and perceptions of food-related services and community interventions in Aberdeen city, with a focus on barriers and facilitators to engagement with these services. Participants (*n* = 20) of different service types, including food, were interviewed between May and July 2024.

A semi-structured topic guide was developed to explore participants’ experiences with food-related services. Questions assessed types of services accessed, how the services operated, how participants accessed them, perceived barriers and facilitators to engagement, and overall satisfaction with the service. Before the interview, participants completed a brief demographic questionnaire capturing age, gender, ethnicity, employment status, monthly income, household composition, and health status. Interviews were conducted in person in private rooms at community centres, and were carried out by AS or OP. All interviews were audio-recorded and transcribed verbatim.

Transcripts were imported into NVivo (version 12) and analysed following Braun and Clarke’s (2006) Six Steps of Reflexive Thematic Analysis. A preliminary codebook was developed by AS, and then checked by JA. Disagreements were settled through discussion. Following this, AS, JA, and OP worked together to identify themes.

#### Positionality statement

AS designed the topic guide for the current study. AS and OP conducted the interviews and AS, JA, and OP conducted the analysis. Two of these authors identify as White-British, and one identifies as White-Romanian. One author has lived experience of food insecurity.

## Results

### Phase 1 – COM-B surveys

Seventy-seven people completed the survey, they were aged from 18 to 86 years (*M*: 45.7, SD: 15.9), evenly split in gender (39F, 38M), with a majority reporting some level of food insecurity. Characteristics of participants are displayed in Table 2.

**Table 2.** Demographic characteristics of participants from survey (phase 1).

Age (mean)	45.7 (±15.9)
Number of people in household (mean)	2.1 (±1.3)
Number of children in household (mean)	0.5 (±0.8)
Gender	
Female	39 (51%)
Male	38 (49%)
Ethnicity	
White British	54 (70%)
White Other	9 (12%)
Black British	1 (1%)
Black Other	8 (10%)
Asian	2 (3%)
Other	3 (4%)
Education	
No education	1 (1%)
Primary school	1 (1%)
Secondary school	28 (36%)
College	24 (31%)
University	20 (26%)
Prefer not to say	3 (4%)
Employment	
Retired	11 (14%)
Student	2 (3%)
Student and working	1 (1%)
Unemployed	37 (48%)
Full time	10 (13%)
Part time	12 (16%)
Volunteers	2 (3%)
Prefer not to say	2 (3%)
Marital status	
Single	38 (49%)
Married	17 (22%0
Living with partner	6 (8%)
Divorced	5 (6%)
In a relationship	5 (6%)
Widowed	4 (5%)
Separated	2 (3%)
Food insecurity (in previous 12 months)	
Concerned about running out of food	57 (74%)
Eaten less than they should have	49 (64%)
Ran out of food	43 (56%)
All of the above *(all due to a lack of money or resources)*	38 (49%)
Engagement with food-related services	46 (60%)
Frequently engaged with services	

The mean scores for each construct, each with a maximum possible score of 5 with 5 representing a high rating of the construct and 1 representing a low rating, are presented in Table 3. Multiple linear regression analysis indicated that capability, opportunity motivation and demographic factors predicted 35.3% of the variance in engagement with food-related services (*F* (6, 64) = 7.377, *p* = 0.001; adj. *R*^2^ = 0.353). Capability (0.438, *p* = 0.011) and opportunity (0.441, *p* = 0.012) were significantly positively associated with engagement indicating that higher levels of self-reported capability and/or opportunity were associated with more frequent engagement with food-related services. Motivation (0.111, *p* = 0.691) was not significantly associated with engagement. Levels of food insecurity, age and gender were also not significantly associated with engagement. Regression coefficients are shown in Table 4.

**Table 3.** Mean scores and standard deviations for each of the COM-B constructs.

Construct	Mean score (/5)	Standard deviation
Capability	3.7	0.9
Opportunity	3.5	0.8
Motivation	3.7	0.6
Behaviour (engagement)	3.4	1.1

**Table 4.** Multiple Linear Regression analysis output from SPSS.

Ratings of capability, opportunity, motivation and behaviour	*B*	SE *B*	*p.*	95% CI for *B*		β	*R* ^2^	Δ*R*^2^
				LB	UB			
Model			0.001*				0.409	0.353
Constant	−0.689	0.927	0.460	−2.541	1.164			
Age	−0.001	0.007	0.935	−0.015	0.014	−0.008		
Gender	0.203	0.224	0.367	−0.244	0.650	0.092		
Food insecurity	0.124	0.104	0.236	−0.083	0.331	0.135		
Capability	0.438	0.167	0.011*	0.105	0.772	0.361		
Opportunity	0.441	0.170	0.012*	0.100	0.781	0.315		
Motivation	0.111	0.279	0.691	−0.446	0.668	0.056		

*B*: unstandardised regression coefficient; CI: confidence interval; LL: lower limit; UL: upper limit; SE *B*: standard error of the coefficient; β: standardised coefficient; *R*^2^: coefficient of determination; Δ*R*^2^: adjusted *R*^2^.

* Significant.

### Phase 2 – Semi-structured interviews

Twenty participants aged between 25 and 65 years (*M*: 46.05, SD: 12.05) took part in the interview component of this study, with 15 identifying as female and 5 as male. The participants’ demographic characteristics are reported in Table 5.

**Table 5.** Demographic characteristics of participants completing interviews.

Participant number	Age range	Gender	Number in household	Number of children in household	Employment status	Marital status	Ethnicity	Highest level of education	Monthly income	Disability reported?
1	35–44	Female	4	3	Looking after household	Single	White (British/Scottish/Irish)	College	<£1200	Yes
2	55–64	Female	1	0	Unemployed	Single	White (British/Scottish/Irish)	None	<£1200	Yes
3	35–44	Female	4	2	Long term sick or disabled	Single	White (British/Scottish/Irish)	College	<£1200	Yes
4	45–54	Female	1	0	Long term sick or disabled	Single	White (British/Scottish/Irish)	School	£1200–£2000	Yes
5	45–54	Female	2	1	Unemployed	Single	White (British/Scottish/Irish)	School	<£1200	No
6	55–64	Female	1	0	Part-time employment	Single	White (British/Scottish/Irish)	College	Prefer not to answer	Yes
7	35–44	Female	4	2	Looking after household	Married/living with partner	White (British/Scottish/Irish)	College	>£2000	No
8	35–44	Female	3	1	Looking after household	Married/living with partner	White (British/Scottish/Irish)	School	>£2000	Yes
9	45–54	Female	1	0	Long term sick or disabled	Divorced/separated	White (British/Scottish/Irish)	None	<£1200	Yes
10	65+	Female	2	0	Retired	Single	White (British/Scottish/Irish)	College	£1200–2000	Yes
11	35–44	Male	2	0	Student	Single	African or African Caribbean	University	<£1200	No
12	25–34	Female	4	2	Looking after household	Married/living with partner	White (British/Scottish/Irish)	School	<£1200	Yes
13	25–34	Male	1	0	Unemployed	Single	White (British/Scottish/Irish)	College	<£1200	Yes
14	25–34	Female	4	3	Looking after household	Divorced/separated	African or African Caribbean	University	<£1200	No
15	65+	Female	2	0	Retired	Married/living with partner	White (other)	None	Prefer not to answer	Yes
16	55–64	Male	1	1	Long term sick or disabled	Divorced/separated	White (British/Scottish/Irish)	School	<£1200	Yes
17	35–44	Male	1	0	Unemployed	Single	White (British/Scottish/Irish)	None	Prefer not to answer	Yes
18	45–54	Female	2	0	Full time carer	Single	White (British/Scottish/Irish)	University	<£1200	No
19	35–44	Female	3	2	Other	Single	White (other)	School	<£1200	No
20	35–44	Male	1	0	Unemployed	Single	White (other)	School	<£1200	No

Four themes, each with subthemes, were identified:

### Theme 1 – Gratitude and reliance

A prominent theme that emerged was the perception of food-related services as a vital lifeline for individuals experiencing food insecurity. Participants expressed deep gratitude for being able to access these services and highlighted how heavily they relied on them to meet basic food needs (subtheme: Gratefulness). This sense of reliance was often accompanied by a reluctance to voice criticism, with participants feeling they should not ‘complain’ given the essential nature of the support. In addition to addressing food needs, participants noted that the services fostered a sense of belonging and social connexion, often facilitating friendships and emotional support (subtheme: Sense of Community).

#### Gratefulness

In this study, participants felt strongly that engaging with food-related services in their local area had improved their lives dramatically and helped them out of difficult situations.

It’s a life saver, it really is. I don’t know what anybody would do without places like this. We would be screwed basically. – PP17

If it wasn’t for the pantry, I’d be lost. – PP15

Gratitude was strongly tied to reliance on services, with participants feeling unable to complain about the services and acknowledging the efforts of providers.

You know it’s slim pickings. One time you’re lucky, the next time you’re not. But again, I’m never complaining I always feel blessed that I get whatever I get. – PP17

Everybody’s trying their best. A food bag’s a food bag. And I shouldn’t really complain about what you get in it. – PP5

#### Sense of community and social support

Participants felt that a key positive outcome of accessing and engaging with services was the social support they received and the friendships they had made.

It’s really nice. It’s like a sense of community. It is, it’s nice. – PP1

. . .when I was introduced to the group I kind of made new friends. . .. -PP9

Participants also referred to these services as a place to meet new people. They spoke about the efforts that other participants would make to ensure new participants felt comfortable and confident to access and engage with these services, feeling welcomed and encouraged.

. . .it was nice to sit there and chat to new people and they kind of really helped encourage me to make use of the services rather than hide away from it. Yeah, they gave me a bit more confidence to use it [. . ..] and we always say to anyone that wants to come along but they’re a bit shy to come and have a chat with us first before we go in so that they don’t feel that they’re not welcome or. . . – PP9

### Theme 2 – Fairness and equity

Despite feeling overwhelmingly grateful to be able to access these services, participants frequently spoke about perceived fairness and equity in the support that they are offered (subtheme ‘Treatment’), provision (subtheme ‘Provision Differs’) and structural inequity in access (subtheme ‘Those most in need’)

#### Treatment

Participants often spoke about their treatment at food-related services and how it impacted their feelings towards the fairness of the service and their desire to engage with it in future. Those with positive experiences felt that those running the services were friendly and helpful

I know they will try their hardest to fit me [. . .] and that’s a nice feeling. It’s a nice feeling that if you were ever properly in need of it, then it would been given. Even if you hadn’t have done the proper channels to get your appointment. It’s a nice feel, it’s nicely run. – PP1

Staff and volunteers were also often described as non-judgemental. Participants felt that they could rely on those running the services to listen to their concerns, treat them fairly and proactively try to help.

They’re really polite. They don’t judge you. They don’t look at you and think oh my god! They’re really helpful actually. – PP8

This support manifested in different ways; sometimes the staff and volunteers helped to ensure participants were getting the food that they need, provided signposting to other more suitable services, and/or advise participants on healthy eating or budgeting.

. . . obviously if you use it every two weeks, they do say well clearly there’s something, do you need some sort of money management or [. . .]and they give you leaflets and say they say, “You’re here every two weeks”, I’m like oh yeah, well seems to be the same. “Is there something you maybe want to look at? [. . .] They will help you. . . – PP8

It is important, however, to acknowledge that not every service was perceived to provide fair and equal treatment to their participants.

You go to some places, they don’t care what you say to them, they kind of shove you aside. – PP2

Some participants felt that there was favouritism within some food banks which resulted in unfair treatment, which subsequently affected their willingness to engage with the service. This sense of unequal treatment conflicted with participants’ expectations of fairness and dignity, particularly given that most people accessing the service were in similarly vulnerable positions

But it’s if your face fits in some of the food banks. Some of them have got favouritism which [there] shouldn’t be. Everybody should get treated the same [. . .] some of the staff like other people more than others and I don’t think that’s right because we’re all there for the same thing – PP6

#### Provision differs between services

Participants spoke of unfair inconsistencies between services and how it influenced their experiences and likelihood to access a service in future. Feelings of unfairness were exacerbated by this sense of a ‘postcode lottery’ where specific services were only accessible to those within a catchment area.

I think it then became postcode specific – PP3

. . . you’ve got to be in a certain area. See if I live in [this area] you can’t go to the one in [that area] or one in another area. . . – PP16

Participants especially felt the effects of the catchment areas when they deemed other areas having better access to and a better choice of services. One participant, for example, spoke about catchment differences in the choice on offer and methods of access (between a large residential pantry vs a small mobile pantry)

It’s just frustrating when you see I’m friends with [REDACTED] and she’s at the [other food pantry] and I know the stuff that they can get and then we have to stand in a car park which is embarrassing [. . .] I think when you know what’s happening in the other pantries and they’re all supposed to be the same [. . .] It’s just a way of, and I think making it fairer, just making sure that all the pantries, if they can, get the same stuff – PP3

#### Those most in need of the services are the least able to access them

Participants highlighted that the logistical issues associated with accessing the services created disproportionate barriers to those most in need, which in turn meant that access to food-related services was not fair or equitable. For example, healthy eating on a budget and cooking classes are often advertised online or via social groups, disadvantaging those most isolated and/or without access to internet. This may mean that those who would benefit most from using these services are the least likely to be exposed to them. Similarly, long walks to food banks, having to stand in long queues, carry heavy items home and having to make appointments at specific times were disproportionately difficult for those with no access to a car, were disabled or had significant mental health issues.

I suppose I went through a phase of never phoning. I have a mental illness which fluctuates and when I’m not on the ball, phoning a pantry the day before to book a space isn’t really top of my list – PP1

If I didn’t have a car, I wouldn’t get here. Know what I mean? I’ve got my car, or I wouldn’t manage. I would be stuck – PP15

But then I think to be fair if I didn’t drive, I wouldn’t be able to go at all because the bags are quite heavy – PP3

It’s all right if you’ve got a pushchair for babies and you can get stuff under your pram and things like that, but I’ve often seen people using their children to carry it because it’s too heavy – PP9

A key example of those most in need having the least opportunity to access was the case of a participant who had recently had a stroke and struggled to use the service

[. . .] it’s difficult because nobody else is allowed to pick up for you [. . .] I’m recovering from a stroke so there’s some weeks that I’m just bedded but nobody else can really pick up for me. They don’t deliver it or there’s, I know they can’t deliver but yeah. – PP3

This participant then often missed out on accessing the food pantry due to post-stroke fatigue and missing their opportunity to visit the pantry, as nobody can visit on their behalf. The pantry only opened for 30 minutes per day, this meant those in work were more likely to miss it, which further builds on this idea that those trying to access work are disadvantaged.

Because even if you’re working or you have anything on, you’re going to miss it – PP3

### Theme 3 - Dignity and stigma

As well as a strong desire for fairness and equity from food-related services, participants also strongly felt that they ought to be treated with dignity (subtheme ‘Differing Standards’), be able to access services discretely (subtheme ‘Privacy and Confidentiality’) and in ways that don’t exacerbate stigma (subtheme ‘Stigmas and Negative Attitudes’).

#### Differing standards

Participants spoke of how food provided was sometimes of low quality, which led to them feeling dehumanised and not treated with dignity. Although grateful to be given food, clients often described receiving short dated or out of date food with limited shelf life.

I don’t know the last time that I actually had like a pack of chicken breasts, or you get things that are already frozen so you’re not quite sure how long they’ve been frozen for, or you’re told that they’ve already been frozen, so you have to use them because they’ve defrosted so you have to use them – PP3

As well as the quality of the food, participants talked about how foods would often be provided in unlabelled packaging. Foods, like meats, would be decanted into food bags and not labelled so participants did not know what kind of meat they were consuming or if it was meat at all. This was problematic when it came to cooking but also exacerbated feelings of dehumanising treatment.

It’s just like burgers in an open packet because they’ve obviously took it out of the main packet. You’re like what is that? – PP8

It’s just a little food bag . . . and I’m like, I don’t even know if it’s chicken or if it’s meat, or if it’s meat free. – PP8

Part of the feeling of indignity comes from the frustration of what participants are given compared to what they would get elsewhere. For example, there would not be an expectation to accept out of date or unlabelled foods from supermarkets or from other food services.

We’re kind of left with the scraps at the end of it. – PP3

#### Privacy and confidentiality

Participants frequently struggled with the lack of privacy and confidentiality in some services, particularly mobile services, offer.

We have to stand in a car park which is embarrassing because everyone thinks you’re just getting a handout [. . .] it’s like a wind tunnel so you’re standing there freezing and there’s like a whole line of 10 or 12 people and everybody can see you – PP3

It’s not even as if you’re hidden from sight but it’s very public. – PP3

Similarly, food services did not have measures in place to protect people’s privacy. For example, participants had to tell staff and volunteers their personal details without a private space to do this.

. . .but everyone that was standing there waiting was like looking you up and down. It’s not really private. – PP7

You’re giving your name and how many are living in your house and your address, people can hear you. That bit I didn’t really like because anyone can take your address – PP17

#### Stigma and negative attitudes

Stigma clearly dissuaded people from accessing services, as they feared what other people would think about them using the service and worried about their negative attitudes.

I found it really hard to go to foodbanks and go to the classes, the eating on a budget classes and healthy eating [. . .] that was quite hard. I was quite embarrassed. I was always worried about what people would say or what people would think. It did put me off for quite a long time . . . – PP9

Soup kitchens, who provide a hot meal to homeless or low-income adults, were spoken about as holding a lot of stigma.

. . .there’s a stigma. All those soup kitchens and stuff like that, I wouldn’t go there even if I was really hungry. I wouldn’t go. I don’t know what it is – PP16

As I said, if you go there, there’s a lot of homeless people that go there and I don’t want to be classed as homeless. There’s a stigma to it, you know? – PP6

Despite this, some participants spoke positively of food pantries and how they combat the stigma that usually arises from using food banks.

It’s to combat the stigma of a food bank so instead of you turning up, how it feels, cap in hand kind of begging for this bag that probably has only half of the things that your family would use because they’re not tailored to you. . . it doesn’t feel like a food bank. You’re just walking in [. . .] “Can I get food parcel please?” and you’re paying for it [. . .] It’s like a poor person shop, that’s exactly what it feels like but better than the stigma of being in a queue of food bank people. Kind of they’re all here just for the same reason. – PP1

### Theme 4 – Mismatch between provision and need

Lastly, the most prominent theme from this analysis was a strong mismatch between provision and need. Participants in this study felt that what the service could offer did not align with what they needed (subthemes ‘Frustration with lack of flexibility, choice’ and ‘Gaps in service capacity and accessibility’).

#### Frustration with lack of flexibility and choice

Frustration was often expressed about the limited flexibility and choice offered by services, which reduced their intentions to engage with them. Many felt that the rigid structures in place did not reflect the realities of their lives or meet their actual needs. This concern was particularly common in relation to food banks, pantries, and soup kitchens, where participants often had little to no input into the contents of the food parcels they received. One of the most common complaints was around the inability to select, substitute or reject food items. As a result, many participants described feeling pressured to take items they did not want, simply to avoid going without.

. . .. they’re getting told if you don’t take this item, you’ll lose it, they’ll take it but then they’re dumping it [. . .] which is silly because somebody could use that – PP7

..if you, say you don’t need cereal you’re not allowed to swap [. . .] You just lose that. – PP3

This inflexibility extended to considerations around household size.

[. . .food parcels] do not increase in size regardless of if the parcel was for a single man or for a large family – PP7

Despite this it is important to recognise that some services offered flexible choice (e.g. allowing participants to take extra for large family or make swaps to accommodate children’s preferences) which was recognised by participants and appreciated.

You get extras along the way if you say, “My kids only eat those kind of crisps.” “Take eight then it’s not a problem” If you’re [not taking a food] because your family doesn’t eat any of this, they’re really good at making sure you come away with more than your £3′s worth. – PP1

Participants also highlighted frustrations at the lack of culturally appropriate food options. Whilst services could cater for vegetarian diets or some specific allergens, food banks and pantries often do not have culturally appropriate foods.

[. . .] because the majority of those who come to these food banks are immigrants, you know, they are not used to the kinds of food you find in the UK, you know? Just for a few. . . they love fruit, they love salad, they love cream, they love milk. But other food, like, pasta, you know? They never had pasta – PP11

The lack of flexibility and choice was often linked with limited usefulness of the food they received. The rigidity of preset food parcels meant that participants were often given ingredients they couldn’t use to create a meal, didn’t want to eat or didn’t know how to prepare, undermining the value of the support they received.

But like basic bags where you’re just getting beans, tomatoes, I get it’s still helping you but it’s not really a meal is it? – PP5

I think services need to look at what they’re providing for them to be able to tick that box and say, yeah we handed out X many parcels this month. – PP15

That’s great for your statistics but how many people actually ate that? They came to you cap-in-hand, hungry and you handed them chickpeas and kidney beans which nobody’s eating that. – PP1

The absence of fresh or meal-building ingredients created added strain.

It’s a lot of tinned soups and a lot of tinned stuff rather than fresh. Pastas and stuff that you can actually make meals with which I think would be a lot better. I think if people were given stuff that actually was available for meals, making meals, I think that would be so much better. – PP9

#### Gaps in service capacity and availability

Participants consistently described a mismatch between when and how services were available and when and how participants were able to access them.

. . . it’s difficult because nobody else is allowed to pick up for you. There’s been some weeks that I can’t get it – PP3

Especially because it’s only [open for] half an hour, so yeah if you miss it for that Thursday [. . .] I’ve got to wait a whole other fortnight for it to come again - PP4

Participants frequently described how limitations in service capacity, including staffing shortages, lack of donations, and restricted resources, directly impacted how often and how easily they could access food support and noted inconsistencies between services:

But [the food bank has] just recently had to go onto fortnightly services because of the shortages [it’s] struggling to get stuff – PP10

. . .they can get theirs weekly and they have two days to pick from. They can go in on a Tuesday or a Thursday . . . They don’t have to stick to the same day. I don’t understand that part. – PP3

## Discussion

Some key factors influencing low-income adults’ engagement with food-related services in areas of deprivation have been identified. The COM-B survey results indicated that individuals perceived that capability and opportunity, but not motivation, positively influenced their engagement with these services. While exploring service participants’ experiences and perceptions revealed that key drivers of [non]engagement related to perceptions of gratefulness, fairness, dignity, stigma, and flexibility in service provision.

User’s perceived capability and opportunity, but not motivation, were associated with service engagement.When individuals perceive greater capability and opportunity, their motivation to engage with a behaviour, such as engaging with services, is likely to increase whereas high motivation alone may not translate into action. In such cases, individuals may feel unable to act, regardless of their intentions. This may explain why motivation had no effect on engagement whilst capability and opportunity did; participants were more likely to engage with services if they had higher ratings of capability and opportunity, but not motivation. This could also suggest that participants may view their ability to engage with services as contingent more on external factors (e.g. structural opportunities and environmental capabilities) than on purely internal drivers like motivation alone. This aligns with some research on health locus of control (Dogonchi et al., 2022), which suggests that individuals with a high internal locus of control (i.e. believe they are personally able to change their health behaviours) are more likely to engage in health-related behaviours. Participants in this study viewed opportunity and capability as more tangible, external barriers than motivation when reflecting on their service use.

Similarly, the positive association between capability and opportunity with engagement is supported by qualitative accounts of how social support, knowing how and where to access support, and feeling fairly treated reduce barriers to using food services. Interestingly, while the survey did not identify motivation as a key factor in engagement, the thematic analysis suggested otherwise; a key facilitator for engaging with food-related services was gratefulness for the service and feeling part of the community, whereas stigmatisation and embarrassment were key barriers. This would suggest that participants who were actively using services were motivated to use them, or vice versa. The PRIME Theory of Motivation, which is complementary to the COM-B model (West and Michie, 2020) may help understand this discrepancy. PRIME goes further than COM-B, and posits that behaviour is driven by planning processes (forming intentions), evaluation processes (weighing pros and cons), motive processes (desiring or rejecting behaviours), and impulse/inhibition processes (acting on or resisting behaviours). Many interviewees described how they planned to use services and had a motive to do so (e.g. to access food or advice). They also evaluated services in terms of its pros and cons (e.g. fairness, dignity, and whether their needs were met), all factors that then influenced their final decision to engage. While participants expressed motivation to use these services, engagement was often restricted by the identified systemic and contextual barriers, highlighting a misalignment between internal drive and external feasibility. It is plausible that perceiving a service as unfeasible may, in turn, reduce an individual’s motivation to engage.

The thematic analysis in the present study also revealed that individuals facing the greatest barriers to accessing services were often the least likely to engage with them, despite arguably standing to benefit the most. This somewhat aligns with the principles of the Inverse Care Law (Fisher et al., 2022; Tudor Hart, 1971), which highlights that those most in need of care are often the least likely to receive it, a pattern frequently observed in primary healthcare settings. In the present study, participants cited logistical barriers, such as limited public transport or lack of access to a car, which hindered their ability to use food-related services and contributed to perceptions of inequitable access. These findings suggest that those who could benefit most from dietary interventions or food service support may be least likely to use them. This highlights the importance of addressing structural barriers to improve the equity and effectiveness of future service delivery.

### Implications

Together the results of the quantitative and qualitative components of this study suggest that engagement with food related services is strongly influenced by perceived ability/opportunities to engage and that these perceptions are related to concrete, and potentially modifiable, facets of service provision such as flexibility and delivery timing. Future interventions should focus on reducing barriers to access, improving perceptions of fairness, and ensuring services align with participants’ needs and preferences.

Thematic analysis of food service user interviews revealed mismatches between service provision and client needs indicating that services may not always meet user expectations. Addressing this issue will be challenging due to funding and staffing constraints, particularly for food banks (Perry et al., 2014). Despite this, future interventions should incorporate target population feedback to ensure they are relevant and effective but most importantly aligned with their needs. Ensuring fairness and dignity in service delivery is a straightforward yet important aim. Services should treat all clients equitably and take steps where practicable to protect clients’ privacy and confidentiality (e.g. by providing secure spaces for sensitive discussions) and avoid stigma (e.g. selecting locations for mobile pantries that are convenient to access but discrete). If food-related services aim to support working families or people living in deprivation, flexibility and accessibility must be central. Narrow operational windows exclude those most in need. These changes may significantly enhance trust and engagement with food-related services, maximising the beneficial impacts they can achieve. These findings should be integrated into future food service delivery models to enhance engagement and accessibility and subsequently tested through rigorous research to determine whether these barriers can be overcome and engagement improved.

### Limitations

A limitation of this study is its reliance on self-reported data about engagement with food-related services. It is possible that non-specific terminology used such as ‘engagement’ may have been interpreted differently by participants as they are open to individual interpretation, making comparisons difficult. Additionally, whilst one question assessed frequency of use, specific details on service engagement (e.g. frequency and number of services used) were not recorded. Including these data would have strengthened the findings and allowed us to draw comparisons between those completing the survey and the interviews.

Another challenge was the wide range of services discussed in interviews. It was initially expected that services would substantially overlap; however, participants described experiences ranging from food banks and pantries (focussed on food provision) to cooking and healthy eating classes (skills and information-based). Thematic analysis was useful in capturing overarching themes, but the diversity of services described made integration challenging. Future research should distinguish between different service types to provide more nuanced insights into how each supports food-insecure individuals.

Finally, the COM-B model may not fully capture the complexity of engagement with food-related services that became apparent from the interview phase of the study. While opportunity and capability account for some external influences, COM-B primarily focuses on individual-level factors and does not fully consider cultural background, structural inequalities, or broader environmental barriers (Whittal et al., 2021). While COM-B offers a broad and flexible framework for understanding behaviour and designing interventions, its application in this study was limited in capturing the nuanced psychological and environmental factors that emerged from participants’ narratives. While participants expressed motivation and a willingness to use food-related services, environmental factors, such as stigma, lack of information, or limited access, often disrupted this.

## Conclusion

This study identifies a complex interplay of factors influencing engagement with food-related services in areas of multiple deprivation. Factors related to capability and opportunity appeared to be most strongly related to engagement, indicating that food-related services and interventions should prioritise strategies that tackle clients’ opportunities and capabilities to access their services.
